# Genomic Prediction of Resistance to Tar Spot Complex of Maize in Multiple Populations Using Genotyping-by-Sequencing SNPs

**DOI:** 10.3389/fpls.2021.672525

**Published:** 2021-07-16

**Authors:** Shiliang Cao, Junqiao Song, Yibing Yuan, Ao Zhang, Jiaojiao Ren, Yubo Liu, Jingtao Qu, Guanghui Hu, Jianguo Zhang, Chunping Wang, Jingsheng Cao, Michael Olsen, Boddupalli M. Prasanna, Felix San Vicente, Xuecai Zhang

**Affiliations:** ^1^Maize Research Institute, Heilongjiang Academy of Agricultural Sciences, Harbin, China; ^2^International Maize and Wheat Improvement Center (CIMMYT), El Batan, Mexico; ^3^College of Agronomy, Henan University of Science and Technology, Luoyang, China; ^4^Maize Research Institute, Anyang Academy of Agricultural Sciences, Anyang, China; ^5^Maize Research Institute, Sichuan Agricultural University, Chengdu, China; ^6^College of Biological Science and Technology, Shenyang Agricultural University, Shenyang, China; ^7^College of Agronomy, Xinjiang Agricultural University, Urumqi, China; ^8^International Maize and Wheat Improvement Center (CIMMYT), Nairobi, Kenya

**Keywords:** maize, tar spot complex, genomic prediction, genomic selection, prediction accuracy, genotyping-by sequencing

## Abstract

Tar spot complex (TSC) is one of the most important foliar diseases in tropical maize. TSC resistance could be furtherly improved by implementing marker-assisted selection (MAS) and genomic selection (GS) individually, or by implementing them stepwise. Implementation of GS requires a profound understanding of factors affecting genomic prediction accuracy. In the present study, an association-mapping panel and three doubled haploid populations, genotyped with genotyping-by-sequencing, were used to estimate the effectiveness of GS for improving TSC resistance. When the training and prediction sets were independent, moderate-to-high prediction accuracies were achieved across populations by using the training sets with broader genetic diversity, or in pairwise populations having closer genetic relationships. A collection of inbred lines with broader genetic diversity could be used as a permanent training set for TSC improvement, which can be updated by adding more phenotyped lines having closer genetic relationships with the prediction set. The prediction accuracies estimated with a few significantly associated SNPs were moderate-to-high, and continuously increased as more significantly associated SNPs were included. It confirmed that TSC resistance could be furtherly improved by implementing GS for selecting multiple stable genomic regions simultaneously, or by implementing MAS and GS stepwise. The factors of marker density, marker quality, and heterozygosity rate of samples had minor effects on the estimation of the genomic prediction accuracy. The training set size, the genetic relationship between training and prediction sets, phenotypic and genotypic diversity of the training sets, and incorporating known trait-marker associations played more important roles in improving prediction accuracy. The result of the present study provides insight into less complex trait improvement via GS in maize.

## Introduction

Tar spot complex (TSC), caused by an interaction of at least three fungal species: *Phyllachora maydis*; *Monographella maydis*; and *Coniothyrium phyllachorae*, is one of the most important foliar diseases of maize (*Zea mays* L. subsp. *mays*) in many Central and South American tropical and subtropical areas (Hock et al., 1992; Pereyda-Hernández et al., 2009). TSC can result in up to 75% grain yield loss, due to reduced ear weight, low kernel filling, and loose kernels. Development and deployment of maize varieties with genetic resistance is the most economical and effective strategy for controlling TSC (Ceballos and Deutsch, 1992).

Understanding the genetic architecture of TSC resistance will allow breeders to improve their breeding efficiency by the implementation of marker-assisted selection (MAS) or genomic selection (GS) to introgress the resistance genes into susceptible germplasm. A few studies have been conducted to dissect the genetic architecture of TSC resistance in maize (Mahuku et al., 2016; Cao et al., 2017). In a collection of 890 inbred lines genotyped with 56 K SNPs, three TSC resistance loci on chromosomes 2, 7, and 8 were identified through association mapping (AM) analysis. The major quantitative resistance locus (QTL) detected on maize chromosome bin 8.03, was furtherly validated in three bi-parental populations through linkage mapping analysis. Identification of the major QTL on bin 8.03 provides the foundation for fine mapping this major QTL and developing functional markers for implementing MAS (Mahuku et al., 2016). The genetic architecture of TSC resistance in maize was confirmed by combined AM and linkage mapping using higher marker density, the major QTL on bin 8.03 was narrowed down to a 33.6 million base pair region, and the results showed that TSC resistance in maize is controlled by a major QTL on bin 8.03, coupled with several minor QTL with smaller effects on other chromosomes (Cao et al., 2017).

Genomic selection is an extension of MAS that uses genome-wide markers to predict the genomic estimated breeding values (GEBVs) of the un-tested lines for selection, where the genome-wide markers are used for selection without detection QTL (Meuwissen et al., 2001; Edriss et al., 2017). In maize, GS has been investigated to improve several major diseases, e.g., maize lethal necrosis resistance (Gowda et al., 2015; Sitonik et al., 2019), northern corn leaf blight resistance (Technow et al., 2013), ear rot resistance (Han et al., 2018; Liu et al., 2020). These studies showed that GS is a promising approach to improve the major diseases, which are under polygenic control. Medium-to-high prediction accuracies were achieved in these studies, and the factors affecting prediction accuracy were assessed over a wide range of target traits. Key factors affecting prediction accuracy include the heritability of the predicted trait (Combs and Bernardo, 2013; Zhang et al., 2015), size of the training set (Zhang et al., 2017), marker density (Spindel et al., 2015), marker quality (Guo et al., 2020), phenotypic, and genotypic variations of the target trait (Gowda et al., 2015), the genetic relationship between training and prediction sets (Isidro et al., 2015; Santantonio et al., 2020; Atanda et al., 2021), and incorporating known trait-marker associations (Bernardo, 2014; Wang et al., 2019), etc. A preliminary genomic prediction analysis has been conducted to investigate the effectiveness of implementing GS for improving TSC resistance in maize, results showed that moderate-to-high prediction accuracies were achieved within different populations using various population sizes and marker densities (Cao et al., 2017). The accuracy of predicting TSC resistance across populations is still unknown under the different factors affecting prediction accuracy.

In the present study, an association-mapping panel and three doubled haploid (DH) populations, genotyped with genotyping-by-sequencing (GBS), were used to estimate the genomic prediction accuracy of TSC resistance in maize. The main objectives of the present study are to: (1) estimate the genomic prediction accuracy of TSC resistance across populations, where the training and prediction sets are different; (2) assess the effect of marker density, marker quality, heterozygosity rate (HT) of samples, the genetic relationship between training and prediction sets, incorporating known trait-marker associations on estimation the genomic prediction accuracy of TSC resistance; (3) explore training population development base on the phenotypic variation of TSC resistance.

## Materials and Methods

### Plant Materials, Phenotyping, and Phenotypic Data Analysis

In the present study, an AM panel and three bi-parental DH populations were used. The AM panel, designated Drought Tolerant Maize for Africa (DTMA) AM panel, consists of 282 tropical and subtropical inbred lines developed by the Global Maize Program of International Maize and Wheat Improvement Center (CIMMYT).

The three DH populations, namely Pop1, Pop2, and Pop3, consists of 174, 100, and 111 lines, respectively. Each of the DH populations was derived from an F_1_ cross formed between a TSC resistant line and a TSC susceptible line, the protocol of generating DH lines was described by Prasanna et al. (2012). The resistant parental lines are widely used CIMMYT maize lines showing good resistance to TSC, and the susceptible parental lines are drought or drought and heat stress-tolerant lines (Yuan et al., 2019) showing severe susceptibility to TSC. The Pop2 and Pop3 shared a common donor line, and the susceptible parental lines of these two populations were derived from the same genetic pool through population improvement. The detailed information of the parental lines was described by Cao et al. (2017).

The DTMA AM panel was evaluated for TSC response in Mexico at five environments, i.e., in Puebla (Latitude: 20°28′; Longitude: −97°38′; Mega environment: lowland tropical) in 2009, 2011 and 2012; in Guerrero (Latitude: 17°02′; Longitude: −99°38′; Mega environment: lowland tropical) in 2012; and in Veracruz (Latitude: 19°15′; Longitude: −96°12′; Mega environment: lowland tropical) in 2012. Pop1 was evaluated for TSC response at three environments, i.e., in Puebla in 2011 and 2014; and in Guerrero in 2013. Pop2 was evaluated for TSC response at four environments, i.e., in Puebla in 2012 and 2014, each year had two planting dates. Pop3 was evaluated for TSC response at three environments, i.e., in Puebla in 2012 with two planting dates; and in Puebla in 2014 (Cao et al., 2017). All the locations used for disease screening had high and consistent natural pressure of TSC. A randomised complete block design was used for all experiments with three replications per location. Each plot consisted of a single 2-m row with 10 plants per row. The TSC score evaluation was performed according to the methods described by Mahuku et al. (2016). The disease severity was recorded using a scale of 1–5 with a 0.5 increment, where 1 = highly resistant (HR), no visible disease symptoms or lesions identifiable on any of the leaves; 5 = highly susceptible (HS), all leaves are dead, no green leaf tissue remaining or disease symptoms on more than 80% of the leaf surface.

MEATA-R software^1^ (Alvarado et al., 2020) was used to analyze multi-location trials using a mixed linear model to estimate the best linear unbiased prediction (BLUP) value of genotypes and the broad-send heritability of the target trait in each population based on the entry mean within trials. The mixed linear model was applied as follows:

$$Y_{i\,j\,k}=\mu +g_{i}+e_{j}+g\,e_{i\,j}+r_{k}\,e_{j}+\varepsilon_{i\,j\,k}$$

where *Y*_*ijk*_ is the target trait, μ is the overall mean, *g*_*i*_, *e*_*j*_, and *ge*_*ij*_ are the effects of the *i*-th genotype, *j*-th environment, and *i*-th genotype by *j*-th environment interaction, respectively. *r*_*k*_*e*_*j*_ is the effect of the *k*-th replication within the *j*-th environment. ε_*ijk*_ is the residual effect of the *i*-th genotype, *j*-th environment, and *k*-th replication. Genotype is considered as the fixed effect, whereas all other terms are declared as the random effects.

Broad-sense heritability (*H*^2^) based on the entry means within trials was estimated as follows:

$$H^{2}=\frac{\sigma_{g}^{2}}{\sigma_{g}^{2}+\frac{\sigma_{g\,e}^{2}}{n\,e}+\frac{\sigma_{e}^{2}}{n\,e\,n\,r}}$$

where $\sigma_{g}^{2}$, $\sigma_{e}^{2}$, and $\sigma_{g\,e}^{2}$ are the genotypic variance, error variance, and genotype-by-environment interaction variance, respectively, and *nr* and *ne* are the numbers of replications and environments, respectively.

### Genotyping and SNP Calling

A commonly used GBS protocol was applied in the present study, which was described in the previous studies (Elshire et al., 2011; Wu et al., 2016; Wang et al., 2020). The SNP calling and imputation was performed according to the methods previously described (Glaubitz et al., 2014; Swarts et al., 2014). Both the un-imputed and the imputed datasets were generated for all four populations of the present study. The un-imputed datasets were only used in the three DH populations to build the block maps to perform linkage mapping analyses. The rest of the analyses were performed with the imputed datasets. Initially, 955,690 SNPs, evenly distributed on the 10 maize chromosomes, were called for each of the genotyped samples.

### Population Structure Analysis

The population structure analysis was performed with the principal components analysis (PCA) in all the four populations, where 232,538 SNPs, filtered with minor allele frequency (MAF) greater than 0.05 and missing rate (MR) less than 20%, were utilised. In the DTMA AM panel, the population structure analysis was applied in software Structure V2.3.3 using an admixture model-based clustering method (Hubisz et al., 2009), where a sub-set of 10,000 SNPs with no missing values were randomly selected to perform this analysis. The heat map of the number of SNPs within 1 Mb physical position was shown in Supplementary Figure 1, which indicates that the 10,000 SNPs almost evenly distribute in 10 maize chromosomes. The average linkage disequilibrium decay distance reported in the previous study was 3.5 Kb at *r*^2^ = 0.1 (Cao et al., 2017). In the DTMA AM panel, evenly distributed SNPs and rapid linkage disequilibrium decay are able to avoid the introduction of bias of oversampling SNPs in the linkage disequilibrium regions in the population structure analysis. Hypotheses were tested for sub-population number *K* ranging from 1 to 10, and each *K* was run seven times with burn-in time and replications both to 100,000.

### Genomic Prediction Analysis

The genomic prediction was implemented in the *rrBLUP* package (Endelman, 2011). In each population, a five-fold cross-validation scheme with 100 replications was used to estimate the prediction accuracy of *r*_*MG*_. The 80% of the lines in each population were randomly assigned as a training set to estimate the effect of the molecular markers and train the prediction model, the rest of the 20% lines were assigned as a validation set in each replication to get the GEBV of each line in the validation set. The average correlation coefficient between the GEBVs and the observed breeding values of the lines in the validation set was defined as the prediction accuracy *r*_*MG*_. Within each of the four populations, SNPs filtered with MAF greater than 0.05 and MR less than 20%, were used for the genomic prediction analyses with a five-fold cross-validation scheme.

### Effect of the Genetic Relationship on the Estimation of the Prediction Accuracy

According to the changes of *ad hoc* statistic delta *K* (Δ*K*) value, the DTMA AM panel was divided into several subgroups. Within each subgroup, a five-fold cross-validation scheme was used to estimate the prediction accuracy of *r*_*MG*_. Besides, the predictions were also conducted between pairwise subgroups, when one subgroup was used as a training set to predict the other subgroup.

Across all the four populations, the predictions were also conducted between pairwise populations, where SNPs filtered with MAF greater than 0.05 and MR less than 20% across all the four populations, were used for the genomic prediction analyses. When the predictions were made across subgroups or populations, the training and validation sets were independent, and the prediction accuracy of *r*_*MG*_ estimated in the validation set was calculated from only a one-time analysis.

### Effect of Marker Quantity and Quality on the Estimation of the Prediction Accuracy

To assess the effect of marker quantity and quality on the estimation of the prediction accuracy, different parameters were applied to filter the SNP dataset within each population to perform the genomic prediction analyses. In each population, a five-fold cross-validation scheme with 100 replications was used to estimate the prediction accuracies. The prediction accuracies were compared, when the SNP datasets filtered with different parameters, were used for genomic prediction analyses.

Different levels of MAF, MR, and HT of the SNPs were used to control the marker quantity and quality. Nine combinations between three MAF levels and three MR levels were used to filter the SNP dataset within each population, MAF setting at 0.05, 0.20, and 0.40; MR setting at 0.00, 10, and 20%. The HT of the SNPs in the DTMA AM panel was set at 1, 3, 5, and 10% after the SNPs were filtered with MAF of 0.05 and MR of 0%, and the prediction accuracies were estimated with a five-fold cross-validation scheme. The effect of the HT of the samples on the estimation of the prediction accuracy was evaluated by setting the HT of the samples at 1, 3, 5, and 10% in the populations of DTMA and Pop1, where the SNPs filtered with MAF greater than 0.05 and MR of 0% were used for prediction analyses. The software of TASSEL V5.0 (Bradbury et al., 2007) was used to filter the imputed dataset with MAF and MR. The customised R scripts were used to filter the HT of the SNPs and samples.

### Genomic Prediction Analyses With the Significantly Associated Markers Detected From the Genetic Mapping

Genomic prediction analyses with significantly associated markers were performed to simulate MAS. In the previous study, 261,948 filtered SNPs were used to perform AM analysis in the DTMA AM panel. In total, 155 SNPs were identified that were significantly associated with TSC resistance in maize at the threshold of −log10 (*P*) > 4.53 (Cao et al., 2017). A five-fold cross-validation scheme was used to assess the accuracies of genomic predictions conducted with the significantly associated markers and the same number of random-selected markers, the number of markers was set as 1, 2, 3, 4, 5, 10, 20, 155, 500, 1000, 3000, 5000, 10,000, 30,000, 50,000, 100,000, and 200,000. The significant markers were selected based on their −log10 (*P*) value, and their chromosome positions. The most significantly associated SNPs were selected on all chromosomes firstly, and then the second significant-associated SNPs were selected.

A block map was constructed in each of the three DH populations to perform linkage mapping in a previous study (Cao et al., 2017), where the blocks were treated as genetic markers to construct the genetic map. In total, 437 blocks in Pop1, 494 blocks in Pop2, and 493 blocks in Pop3 were built with 20,473, 27,818 and 326,07 SNPs, respectively. In the software of QTL IciMapping Version 4.1 (Meng et al., 2015), the single-marker analysis method was used to perform the linkage mapping analyses and rank the scores of the log of the odds of all the blocks, the scores of the log of the odds representing the significant levels of the association between the block and the TSC resistance. A five-fold cross-validation scheme was used to assess the accuracies of genomic predictions conducted with the significantly associated markers and the same number of random-selected markers, the number of markers was set as 5, 10, 15, 20, 30, 50, 100, 200, 300, 400, and all the blocks in each population.

In the above analyses, the prediction accuracy could be overestimated, because the same population was used to identify the significantly associated markers firstly, and then it was used to calculate the prediction accuracy estimated with the significantly associated markers. To avoid the overestimated prediction accuracy, the 150 significantly associated markers detected from the DTMA AM panel were used for estimating the prediction accuracy in each of the three DH populations, when the DTMA AM panel was used as the training set, and the DH population was used as the validation set. For comparison, 150 randomly selected markers were also used to estimate the prediction accuracy in each of the three DH populations.

### Training Set Development Based on the Phenotypic Variation of TSC Resistance

According to the phenotypic variation information of the TSC resistance in each population, training sets were formed. Four scenarios were simulated and compared within each of the four populations, where the training set was formed by sampling the same percentage of materials with a selection from both resistant and susceptible tails (R + S), with random selection (RD), with a selection from the resistant tail (R), with a selection from the susceptible tails (S), respectively. In each scenario, the validation set was the whole population, and the training set ranged from 20 to 60%, with an interval of 20%. In each of the four populations, a total of 12 combinations and comparisons were conducted between the four scenarios and the three percentage levels of the training set.

## Results

### Phenotypic Variation, Heritability, and Phenotypic Correlation Between Locations

The BLUP value of TSC resistance of all the genotypes across the four populations ranged from 1.31 to 4.39. The Pop3 had the widest range of variation among the four populations. The minimum BLUP value was 1.31,1.81, 1.18, and 1.37 in the DTMA AM panel, Pop1, Pop2, and Pop3, respectively. The maximum BLUP value was 3.23, 3.00, 3.95, and 4.39 in the DTMA AM panel, Pop1, Pop2, and Pop3, respectively. The heritability of TSC resistance across locations was 0.80, 0.54, 0.88, and 0.93 in the DTMA AM panel, Pop1, Pop2, and Pop3, respectively. The average phenotypic correlation coefficient of TSC resistance between locations was 0.47, 0.37, 0.68, and 0.84 in the DTMA AM panel, Pop1, Pop2, and Pop3, respectively.

### Population Structure Analysis Within and Among Populations

**FIGURE 1:**
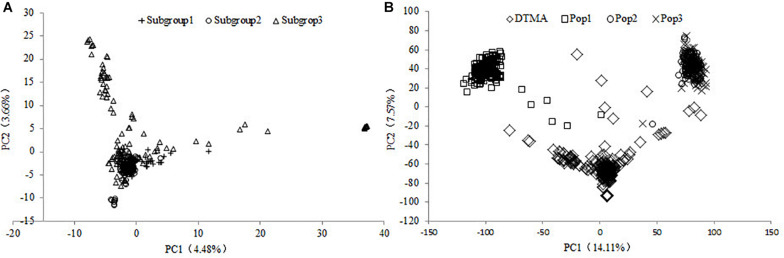
Results of the principal components (PC) analysis in the **(A)** DTMA association mapping panel, and in **(B)** all the four populations of DTMA association mapping panel, Pop1, Pop2, and Pop3.

According to the *ad hoc* statistic Δ*K* value changes, the DTMA AM panel was divided into three subgroups, the number of lines was 40, 111, and 131 in Subgroups 1, 2, and 3, respectively (Cao et al., 2017). Most of the lines in Subgroup 1 were from the Mexico physiology research group, lines in Subgroup 2 were mainly from the subtropical breeding program, and lines in Subgroup 3 were mainly from the lowland tropical breeding program. The result of the structure split for all the Ks (1–10) was provided in Supplementary File 1. The population structure within the DTMA AM panel was illustrated with the first two principal components in Figure 1A, where the results showed the first and second principal components explained 4.48 and 3.66% of the total SNP variation, respectively. Some lines from each subgroup centrally clustered with each other, indicating the moderate level of genetic relatedness among the subgroups. The inbred lines in the Subgroup 3 were most widely scattered, implying the broadest genetic diversity presented in Subgroup 3 among all the three subgroups. These observations are consistent with the current germplasm exchange patterns where there is a constant flow of germplasm among the subgroups.

The genetic relationship among the four populations was illustrated with the first two principal components in Figure 1B, where the results showed the first and second principal components explained 14.11 and 7.57% of the total SNP variation, respectively. The inbred lines in the DTMA AM panel were most widely scattered, implying the broadest genetic diversity presented in the DTMA AM panel among all four populations. The DTMA AM panel was not overlapped with any of the three bi-parental populations, the Pop1 was not overlapped with either the Pop2 or the Pop3. The Pop2 was overlapped with the Pop3, due to the common parent shared by these two populations, it indicated the closest relationships between these two populations.

### Genomic Prediction Accuracies Obtained Within and Across Populations and Subgroups

Genomic prediction accuracies obtained from five-fold cross-validations and 100 replications were high in all four populations, when the SNP datasets, filtered with MAF greater than 0.05 and MR less than 20%, were used to perform prediction within each population. The number of SNPs after filter in the DTMA AM panel, Pop1, Pop2, and Pop3 were 261,948, 98,018, 102,204, and 104,046, respectively. The *r*_*MG*_ values observed in the DTMA AM panel, Pop1, Pop2, and Pop3 were 0.56, 0.60, 0.75, and 0.69. The *r*_*MG*_ value observed in the DTMA AM panel was lower than those observed in the DH populations.

Genomic prediction accuracies obtained from five-fold cross-validations and 100 replications were low to moderate within the three subgroups of the DTMA AM panel (Table 1). The *r*_*MG*_ values observed in the Subgroup 1, Subgroup 2, and Subgroup 3 were 0.27, 0.55, 0.35, respectively. The *r*_*MG*_ values observed in the subgroups of the DTMA AM panel were lower than those observed in the DTMA AM panel.

**TABLE 1:** Genomic prediction accuracies for TSC resistance obtained between the three subgroups of the DTMA association mapping panel.

Training set (number of lines)	Validation set	Prediction accuracy
Subgroup 1 (40)	Subgroup 1	0.27
	Subgroup 2	−0.08
	Subgroup 3	−0.03
Subgroup 2 (111)	Subgroup 2	0.55
	Subgroup 1	−0.3
	Subgroup 3	0.07
Subgroup 3 (131)	Subgroup 3	0.35
	Subgroup 1	0.16
	Subgroup 2	0.33

Genomic prediction accuracies obtained across subgroups were relatively low when the predictions were performed between pairwise subgroups (Table 1). The *r*_*MG*_ values observed between pairwise subgroups ranged from −0.30 to 0.33, the relative high prediction accuracies were observed, when Subgroup 3 was used as a training set to predict the other two subgroups, because of the bigger population size and broadest genetic diversity presented in Subgroup 3 contributing to the improvement of prediction accuracy. The *r*_*MG*_ values observed between pairwise subgroups were lower than those observed within the subgroups.

Genomic prediction accuracies obtained across populations varied in different scenarios and ranged from 0.20 to 0.64 (Table 2). The plots of the correlation between the predicted and the observed BLUP values for these predictions were shown in Supplementary Figure 2. When the DTMA AM panel was used as the training set, the *r*_*MG*_ values observed in the Pop1, Pop2, and Pop3 were 0.45, 0.61, and 0.55, respectively. When the DH populations were used as the training set to predict the DTMA AM panel, the *r*_*MG*_ values observed in the DTMA AM panel were relatively low and ranged from 0.20 to 026. The *r*_*MG*_ values observed between the pairwise DH populations were moderate to high and ranged from 0.36 to 0.64. The highest *r*_*MG*_ values were observed in pairwise populations of Pop2 and Pop3, i.e., 0.64 and 0.60. The lowest *r*_*MG*_ values were observed in pairwise populations of Pop1 and Pop3, i.e., 0.36 and 0.40.

**TABLE 2:** Genomic prediction accuracies for TSC resistance obtained between all the four populations of DTMA association mapping panel, Pop1, Pop2, and Pop3.

Training set (number of lines)	Validation set	Prediction accuracy
DTMA (282)	Pop1	0.45
	Pop2	0.61
	Pop3	0.55
Pop1 (174)	DTMA	0.26
	Pop2	0.61
	Pop3	0.40
Pop2 (100)	DTMA	0.20
	Pop1	0.52
	Pop3	0.60
Pop3 (111)	DTMA	0.23
	Pop1	0.36
	Pop2	0.64

### Genomic Prediction Accuracies Obtained From Different Levels of Marker Density, Marker Quality, and Heterozygosity Rate of Samples

Across all the populations, the number of markers decreased as the MAF increased and the MR decreased, the marker quality improved as the number of markers decreased. The maximum number of markers and the highest MD were observed by filtered the SNPs with the combination of MAF of 0.05 and MR of 20%, the minimum number of markers and the lowest MD were observed by filtered the SNPs with the combination of MAF of 0.40 and MR of 0%. The number of SNPs filtered with the combination of MAF of 0.05 and MR of 20% in the DTMA AM panel, Pop1, Pop2, and Pop3 was 261,948, 98,018, 102,204, and 104,046, respectively. The number of SNPs filtered with the combination of MAF of 0.40 and MR of 0% in the DTMA AM panel, Pop1, Pop2, and Pop3 was 1144, 61,471, 65,923, and 61,525, respectively.

**FIGURE 2:**
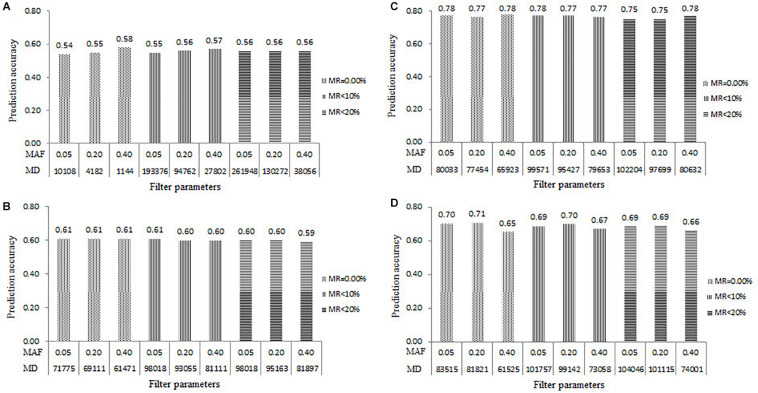
Genomic prediction accuracies for TSC resistance estimated from the five-fold cross-validation scheme in all the four populations of **(A)** DTMA association mapping panel, **(B)** Pop1, **(C)** Pop2, and **(D)** Pop3, under the nine levels of marker density (MD) filtered with the combinations of three levels of minor allele frequency (MAF) and three levels of missing rate (MR).

The prediction accuracy results estimated in all the four populations under the nine marker datasets filtered with the combinations of MAF and MR were shown in Figure 2. Within each population, the *r*_*MG*_ values estimated with the different marker datasets were slightly different. The *r*_*MG*_ values ranged from 0.54 to 0.58 in the DTMA AM panel, from 0.59 to 0.61 in the Pop1 population, from 0.75 to 0.78 in the Pop2 population, and from 0.65 to 0.71 in the Pop3 population. Across all the populations, relatively high and similar prediction accuracies were obtained across all levels of MAF and MR, indicating that the levels of MAF and MR had minor effects on the estimation of the prediction accuracy.

**FIGURE 3:**
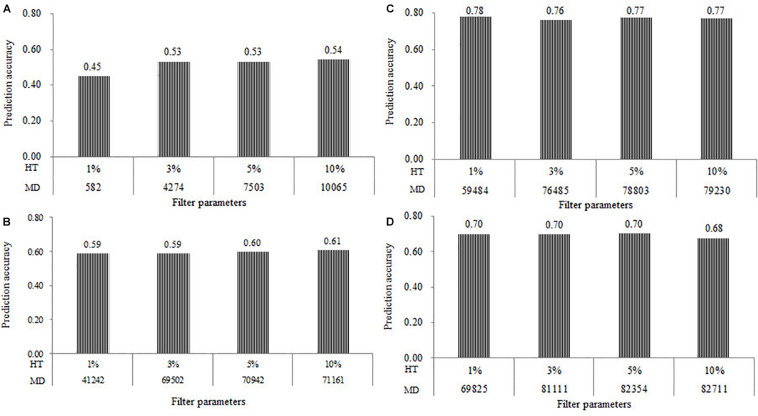
Genomic prediction accuracies for TSC resistance obtained in the **(A)** DTMA association mapping panel, **(B)** Pop1; **(C)** Pop2; **(D)** Pop3, under the different levels of marker density (MD) at the four levels of heterozygosity rate (HT) of SNPs at 1, 3, 5, and 10%, and filtered with the combination of minor allele frequency (MAF) of 0.05 and missing rate (MR) of 0%.

The prediction accuracy results of all the four populations estimated at the four levels of HT of SNPs at 1, 3, 5, and 10% were shown in Figure 3. Under the combination of MAF of 0.05 and MR of 0%, the number of markers in the DTMA AM panel filtered with the HT of SNPs at 1, 3, 5, and 10% were 582, 4274, 7503, and 10,065, respectively. The *r*_*MG*_ values estimated from the number of SNPs of 582, 4274, 7503, and 10,065 were 0.45, 0.53, 0.53, and 0.54, respectively (Figure 3A). A significant increase of the *r*_*MG*_ value was observed in the DTMA AM panel, when the HT of SNPs changed from 1 to 3% and the number of SNPs increased from 582 to 4274. Under the combination of MAF of 0.05 and MR of 20%, the number of markers in all the DH populations were filtered with the HT of SNPs at 1, 3, 5, and 10%. The slight differences were observed on the *r*_*MG*_ values, as the HT of SNPs increased in all the DH populations (Figures 3B–D). These results indicated that the effect of HT of SNPs on the estimation of the prediction accuracy is mainly caused by the changes in the number of SNPs.

**FIGURE 4:**
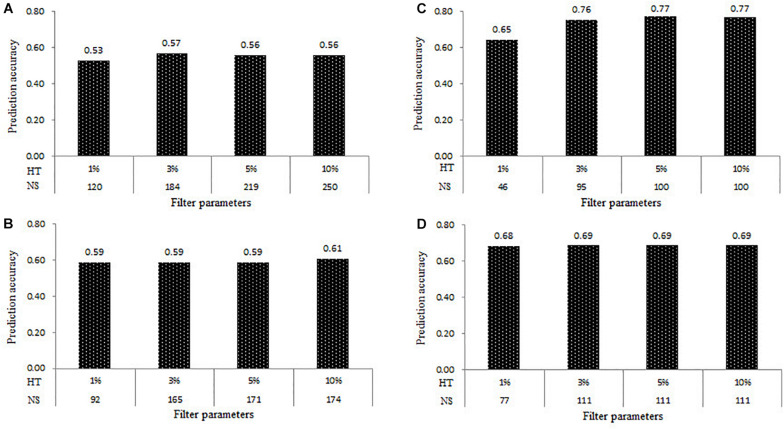
Genomic prediction accuracies for TSC resistance obtained in the four populations of the **(A)** DTMA association mapping panel, **(B)** Pop1, **(C)** Pop2, and **(D)** Pop3, at the four levels of heterozygosity rate (HT) of samples of 1, 3, 5, and 10%, and the different number of samples (NS).

The prediction accuracy results of all the four populations estimated at the four levels of HT of samples at 1, 3, 5, and 10% were shown in Figure 4. Under the combination of MAF of 0.05 and MR of 0%, the number of samples in the DTMA AM panel filtered with the HT of the sample at 1, 3, 5, and 10% were 120, 184, 219, 250, respectively. The *r*_*MG*_ values estimated in the DTMA AM panel at the HT of samples of 1, 3, 5, and 10% were 0.53, 0.57, 0.56, and 0.56, respectively. In Pop1, the number of samples filtered with the HT of samples at 1, 3, 5, and 10% was 92, 165, 171, and 174, respectively. The *r*_*MG*_ values estimated in Pop1 at the HT of samples of 1, 3, 5, and 10% were 0.59, 0.59, 0.59, and 0.61, respectively. In Pop2, the number of samples filtered with the HT of samples at 1, 3, 5, and 10% was 46, 95, 100, and 100, respectively. The *r*_*MG*_ values estimated in the Pop2 at the HT of samples of 1, 3, 5, and 10% were 0.65, 0.76, 0.77, and 0.77, respectively. In the Pop3, the number of samples filtered with the HT of samples at 1, 3, 5, and 10% was 77, 111, 111, and 111, respectively. The *r*_*MG*_ values estimated in Pop3 at the HT of samples of 1, 3, 5, and 10% were 0.68, 0.69, 0.69, and 0.69, respectively. Similar trends were observed across all four populations, the slight increases were observed on the *r*_*MG*_ values, as the HT of samples increased. These results showed that the effect of HT of samples on the estimation of the prediction accuracy is mainly caused by the changes in the number of samples.

### Genomic Prediction Accuracies Obtained From the Significantly Associated Marker

**FIGURE 5:**
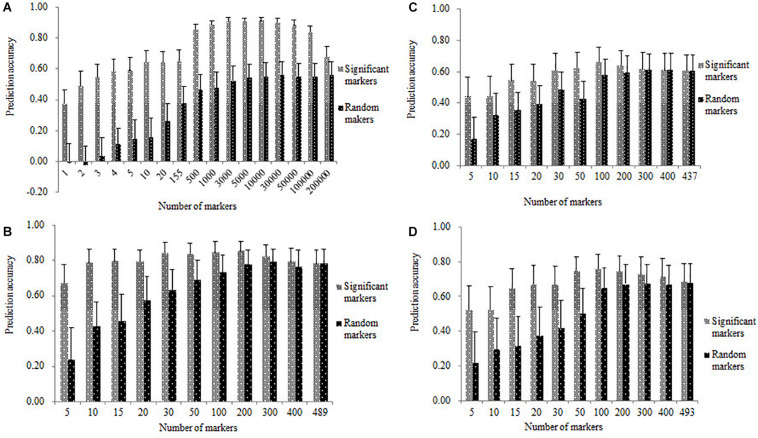
Genomic prediction accuracies for TSC resistance estimated with the same number of significant and random markers in all the four populations of **(A)** DTMA association mapping panel, **(B)** Pop1, **(C)** Pop2, and **(D)** Pop3.

Genomic prediction accuracies in all the four populations estimated with the significantly associated SNPs were shown in Figure 5, where relatively high *r*_*MG*_ values were obtained with a few significantly associated markers in each of the four populations. The *r*_*MG*_ values obtained from the significantly associated SNPs were consistently higher than those obtained from the same number of randomly selected markers. In the DTMA AM panel, the number of significant-associated markers detected on the chromosomes of 2, 3, 7, and 8, were 1, 3, 1, and 150, respectively. The significantly associated SNPs used for prediction were ranked based on the information of their significant *p*-values and physical positions, and the top five significantly associated SNPs with the lowest *p*-values used for prediction were selected from the chromosomes of 8, 3, 2, 7, and 3, respectively. In the DTMA panel, the *r*_*MG*_ value obtained from the most significantly associated SNP on chromosome 8 was 0.37. The *r*_*MG*_ values obtained from the top two, top three, top four, and top five significantly associated SNPs were 0.49, 0.54, 0.58, and 0.59, respectively. The *r*_*MG*_ values consistently increased, as more significantly associated SNPs were used for prediction. The *r*_*MG*_ values reached the plateau, once the number of significantly associated SNPs used for prediction increased to more than 500. Similar trends were observed in the three DH populations, the *r*_*MG*_ values obtained from the significantly associated markers were consistently higher than those obtained from the same number of randomly selected markers, the *r*_*MG*_ values reached the plateaus in the DH populations, once the number of significantly associated markers used for prediction increased to more than 50. These results indicated that incorporating the significantly associated SNPs into GS has the potential for improving the prediction accuracy.

Genomic prediction accuracies in the DH populations of Pop1, Pop2, and Pop3 estimated with the 150 significantly associated SNPs were higher than those estimated with the same number of randomly selected SNPs (Table 3), when the DTMA AM panel was used as the training set to predict the DH population as the validation set. The genomic prediction accuracies estimated with the 150 significantly associated SNPs were 0.39, 0.49, and 0.43 in the Pop1, Pop2, and Pop3, respectively. The genomic prediction accuracies estimated with the 150 randomly selected SNPs were 0.09, 0.15, and 0.11 in the Pop1, Pop2, and Pop3, respectively.

**TABLE 3:** Genomic prediction accuracies in the DH populations of Pop1, Pop2, and Pop3 estimated with the 150 significantly associated SNPs and the same number of randomly selected SNPs.

Training set	Validation set	Prediction accuracy estimated with the 150 significantly associated SNPs	Prediction accuracy estimated with the 150 randomly selected SNPs
DTMA	Pop1	0.39	0.09
DTMA	Pop2	0.49	0.15
DTMA	Pop3	0.43	0.11

### Training Set Development Based on the Phenotypic Variation of the Target Trait

**FIGURE 6:**
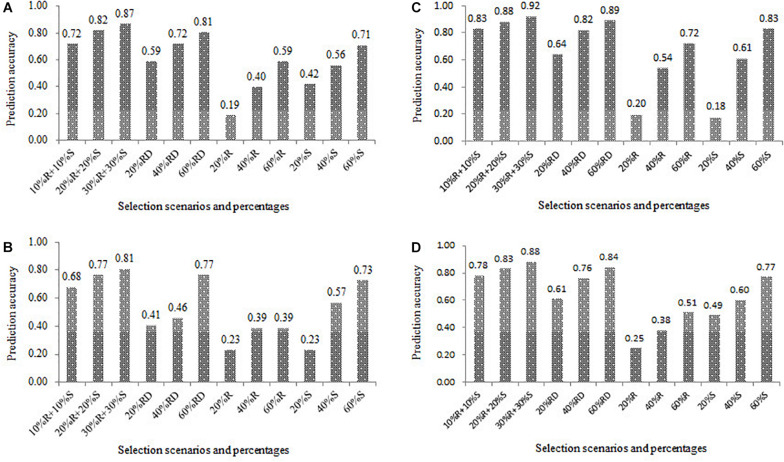
Genomic prediction accuracies estimated in the 12 combinations between the four scenarios and the three percentage levels of the training set (20, 40, and 60%) in the four populations of **(A)** DTMA association mapping panel, **(B)** Pop1, **(C)** Pop2, and **(D)** Pop3. The scenario of R + S represents the selection from both resistant and susceptible tails, RD represents the random selection, R represents the selection from the resistant tail, S represents the selection from the susceptible tail.

For all the four populations, the results of the prediction accuracies estimated in the 12 combinations between the four scenarios and the three percentage levels of the training set were presented in Figure 6. Across all four scenarios, the prediction accuracy increased in all the populations as the increase of percentage of the training set. For example, the prediction accuracies in the scenario of R+S were 0.72, 0.82, and 0.87, when the percentages of the training set in the DTMA panel were set as 20, 40, and 60%, respectively. Under the same percentage of the training set, the scenario of R+S outperformed the other three scenarios, and the scenario of RD outperformed the other two scenarios of R and S. For example, the prediction accuracy in the DTMA panel at the percentage of the training set at 60% were 0.87, 0.81, 0.59 and 0.71 for the scenario of R+S, RD, R, and S, respectively. Similar trends were also observed in the three DH populations. These results indicated that the training set development with broad phenotypic variation has the potential improving prediction accuracy.

## Discussion

In tropical and subtropical areas of Central and South America, TSC is one of the most destructive foliar diseases of maize, it may cause up to 75% grain yield loss. A few genetic studies have been conducted to dissect the genetic architecture of resistance to TSC of maize (Mahuku et al., 2016; Cao et al., 2017), where the heritabilities of TSC in different populations were medium-to-high, revealing that the phenotypic selection is effective for improving TSC resistance. However, improving TSC resistance through phenotypic selection is cost-intensive and time-consuming, because multiple location trials are required to improve TSC resistance through phenotypic selection.

Previously published studies revealed that TSC resistance in maize is controlled by a major QTL on bin 8.03, coupled with several minor QTL with smaller effects on other chromosomes. Fine mapping the major QTL on bin 8.03 and developing function markers associated with this major QTL will facilitate the implementation of MAS for improving breeding efficiency, and saving cost. In the present study, the effectiveness of MAS was simulated, when a few significantly associated SNPs were used for GS. In the DTMA panel, the prediction accuracy estimated with the most significantly associated SNPs on bin 8.03 was 0.37, and the prediction accuracy continuously increased as more significantly associated SNPs were used for GS. A similar trend was also observed in the three DH populations. These results implied that it is effective to improve the TSC resistance in maize by implementing MAS for introgression of the major QTL on bin 8.03 into susceptible germplasm. Moreover, TSC resistance in tropical maize could be furtherly improved by implementing GS for selecting multiple stable genomic regions simultaneously, or by implementing MAS and GS stepwise.

In maize, GS has been shown as an effective genomic tool to improve breeding efficiency and accelerate genetic gain over a wide range of target traits with different levels of genetic complexity (Crossa et al., 2017). GS was implemented in various kinds of the population to estimate the genomic prediction accuracy of different target traits in several previous studies (Zhao et al., 2012; Vélez-Torres et al., 2018). In the previous study, moderate-to-high prediction accuracies of TSC resistance were achieved within each of the four populations (Cao et al., 2017). In the present study, moderate-to-high prediction accuracies were achieved across populations by using the training sets with broader genetic diversity, and in pairwise populations having closer genetic relationships. These results implied that a collection of inbred lines with broader genetic diversity could be phenotyped in multiple locations and used as a permanent training set, which will be employed to implement GP on the untested new populations. The training set could be updated by incorporating more new phenotyped lines, which have closer genetic relationships with the prediction set. Therefore, higher prediction accuracies can be achieved by strengthening the genetic relationship between the training and prediction sets and increasing the size of the training set (Riedelsheimer et al., 2013). This strategy will enhance breeding efficiency and save costs dramatically for improving TSC resistance in a breeding program. Moreover, a common training set also could be built for the implementation of GS on multiple traits improvement, especially for the less complex traits of foliar diseases or nutritional quality traits in maize, which can be predicted very well by using a collection of inbred lines with broad genetic diversity as the training set.

Implementation of GS requires a profound understanding of factors affecting genomic prediction accuracy (Zhang et al., 2017). In the previous study, the effects of training set size and marker density on the estimation of the genomic prediction accuracy of TSC resistance were investigated (Cao et al., 2017). In the present study, the effects of factors of marker density, marker quality, HT of samples, phenotypic diversity of the training set, incorporating known trait-marker associations on the estimation of the genomic prediction accuracy of TSC resistance were furtherly assessed. Results showed that the levels of MAF, MR, and HT of SNPs had minor effects on the estimation of the prediction accuracy. The effects of MAF, MR, and HT of SNPs on the estimation of the prediction accuracy are mainly caused by the changes in the number of SNPs. Once the number of SNPs is saturated on each chromosome, and at least one SNP per linkage disequilibrium block is selected for prediction, the prediction accuracy reaches a plateau (Lorenzana and Bernardo, 2009). There is a tradeoff between the number of markers and marker quality, marker quality becomes lower as the number of markers increases in a specific marker dataset. Appropriate levels of MAF, MR, and HT of SNPs should be considered and selected to improve the prediction accuracy and reduce the computational burden by balancing the number of markers and marker quality, this result is consistent with several previous studies (Guo et al., 2020). Within each of the four populations, slight increases in prediction accuracy were also observed, as the HT of samples increased and the training set size enlarged, indicating that training set size is an important factor improving prediction accuracy (Combs and Bernardo, 2013).

Selective genotyping is proposed to improve QTL mapping and save cost in bi-parental populations, where only the individuals from one or two tails with extreme phenotypic values are genotyped (Sun et al., 2010). In the present study, the R + S scenario built the training set by selecting the individuals from two tails with extreme phenotypic values, the R + S scenario had higher prediction accuracies than those in other scenarios. Taking the advantages of more accurate phenotyping and abundant phenotypic variation, the R + S scenario outperformed other scenarios. It implies that prediction accuracy can be improved by developing a training set with broad phenotypic variation, as well as broad genotypic diversity indicated in several previous studies (Gowda et al., 2015; Guo et al., 2020).

## Data Availability Statement

The original contributions presented in the study are publicly available. These data can be found at the CIMMYT Research Data & Software Repository Network: https://hdl.handle.net/11529/10548579.

## Author Contributions

BP, MO, FS, and XZ conceived and designed the overall study. FS and XZ coordinated the phenotyping. BP, MO, and XZ coordinated the genotyping. SC, JS, YY, AZ, JR, YL, JQ, GH, JZ, CW, and JC analysed the data. SC, JS, FS, and XZ drafted the manuscript. SC, JS, MO, BP, FS, and XZ interpreted the results. All authors contributed to the manuscript editing. All authors contributed to the article and approved the submitted version.

## Conflict of Interest

The authors declare that the research was conducted in the absence of any commercial or financial relationships that could be construed as a potential conflict of interest.

## Footnotes

1 http://hdl.handle.net/11529/10201
