# Supplementary material for: Genomic Prediction of Resistance to Tar Spot Complex of Maize in Multiple Populations Using Genotyping-by-Sequencing SNPs
Source: Front Plant Sci. 2021 Jul 16;12:672525. doi: 10.3389/fpls.2021.672525 (PMC8322742; doi:10.3389/fpls.2021.672525)
Supplement: Supplementary file 2 [file Data_Sheet_2.zip › Figure S1.docx]

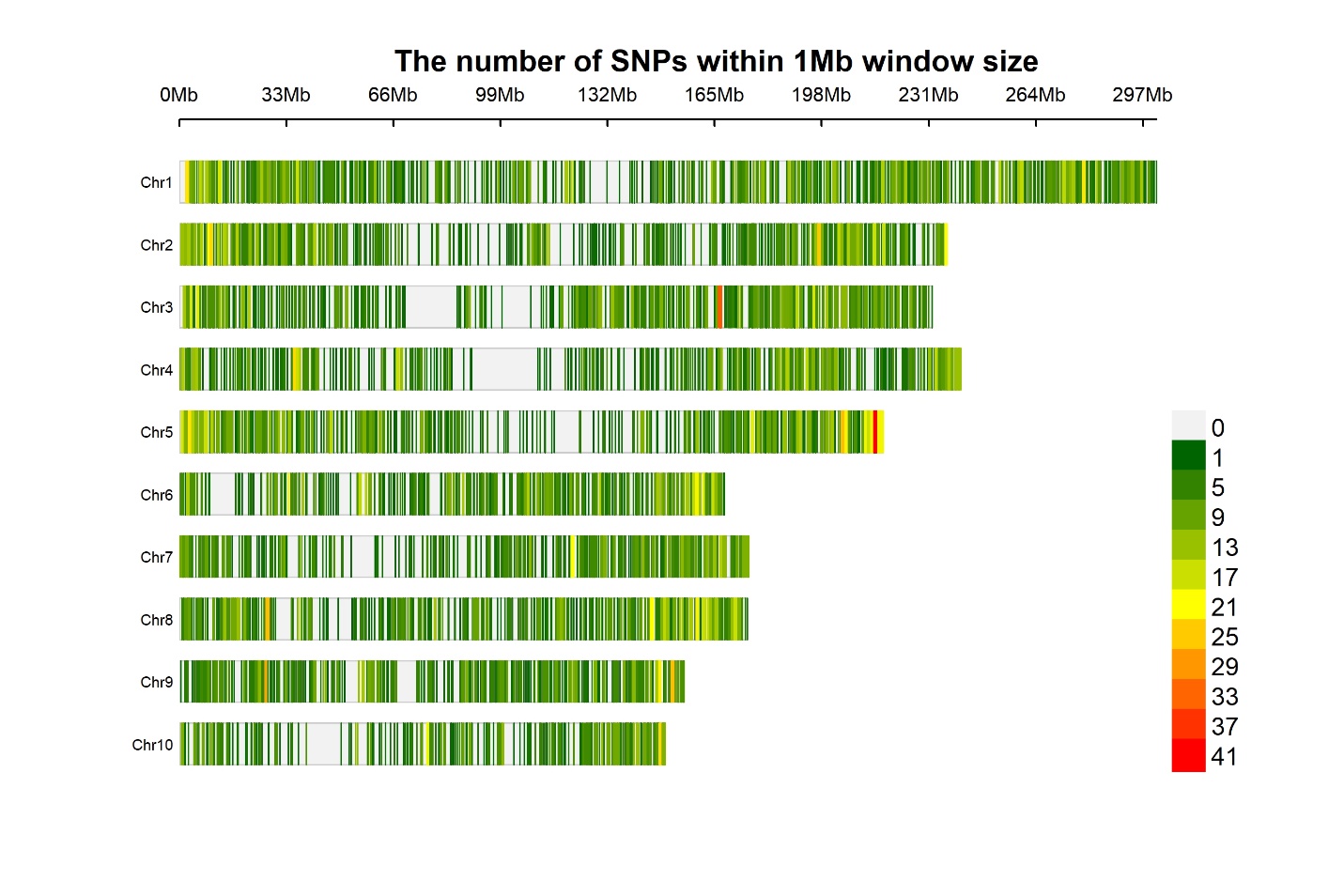


Figure S1 The heat map of the number of SNPs within 1 Mb physical position for the 10,000 SNPs used for population structure analysis.
