# Supplementary material for: Genomic Prediction of Resistance to Tar Spot Complex of Maize in Multiple Populations Using Genotyping-by-Sequencing SNPs
Source: Front Plant Sci. 2021 Jul 16;12:672525. doi: 10.3389/fpls.2021.672525 (PMC8322742; doi:10.3389/fpls.2021.672525)
Supplement: Supplementary file 2 [file Data_Sheet_2.zip › Figure S2.docx]

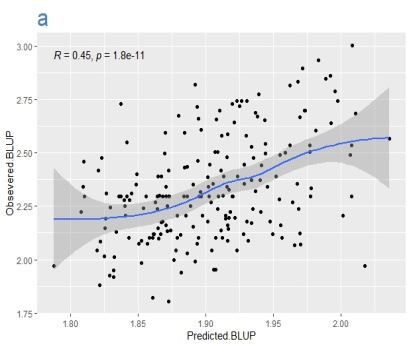

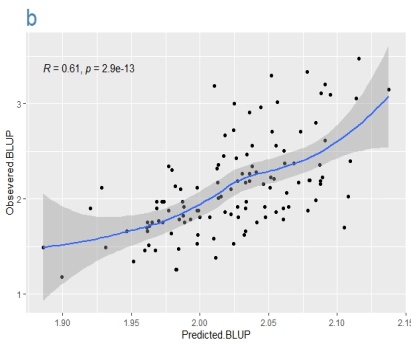

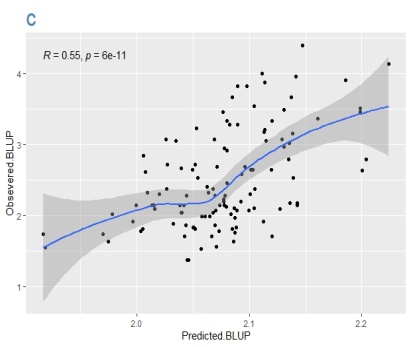


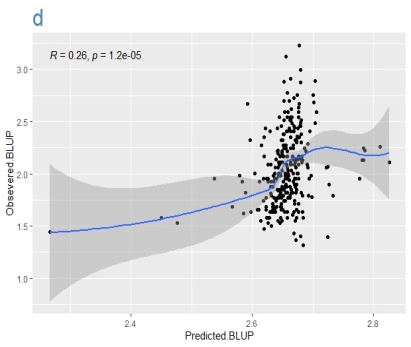

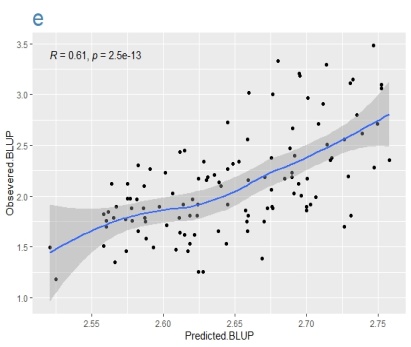

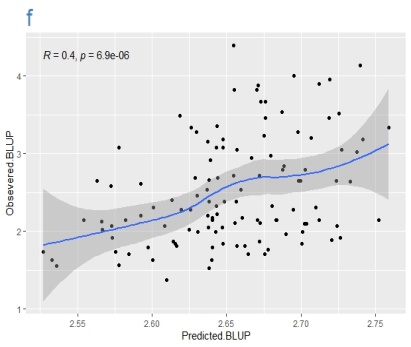

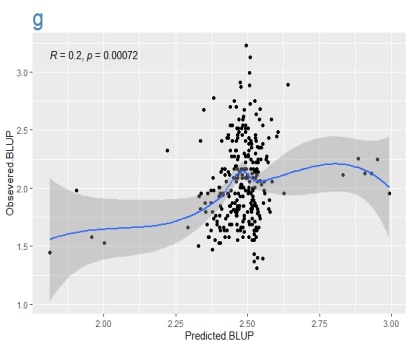

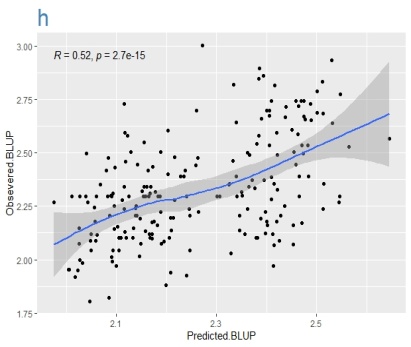

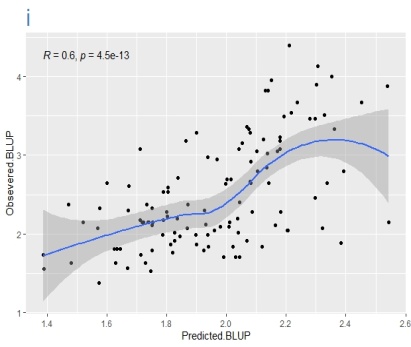

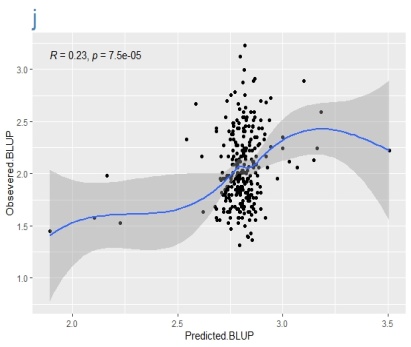

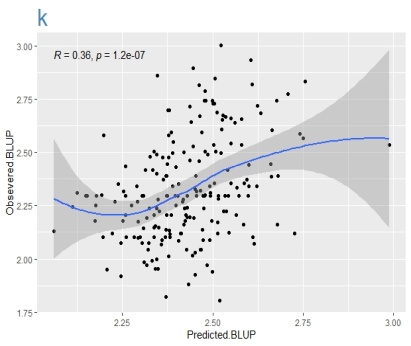

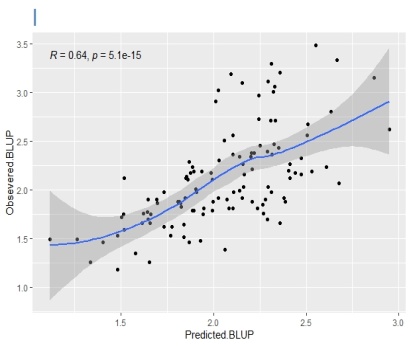


Figure S2 Plots of the correlation between the predicted and the observed BLUP values for all the predictions made in Table 2, when the training and validation sets were different, and the predictions were performed across populations.

(a) DTMA AM panel predicted Pop1; (b) DTMA AM panel predicted Pop2; (c) DTMA AM panel predicted Pop3; (d) Pop1 predicted DTMA AM panel; (e) Pop1 predicted Pop2; (f) Pop1 predicted Pop3; (g) Pop2 predicted DTMA AM panel; (h) Pop2 predicted Pop1; (i) Pop2 predicted Pop3; (j) Pop3 predicted DTMA AM panel; (k) Pop3 predicted Pop2; (l) Pop3 predicted Pop2.
